# Effect of competitive cues on reproductive morphology and behavioral plasticity in male fruitflies

**DOI:** 10.1093/beheco/arv170

**Published:** 2015-10-25

**Authors:** Amanda Bretman, Claudia Fricke, James D. Westmancoat, Tracey Chapman

**Affiliations:** ^a^School of Biology, University of Leeds, Manton Building, Leeds LS2 9JT, UK,; ^b^Institute for Evolution and Biodiversity, University of Muenster, Huefferstr. 1, 48149 Muenster, Germany, and; ^c^School of Biological Sciences, University of East Anglia, Norwich Research Park, Norwich NR4 7TJ, UK

**Keywords:** accessory gland, behavioral plasticity, developmental plasticity, larval density, social and sexual environment, testis.

## Abstract

Phenotypic plasticity will be favored whenever there are significant fitness benefits of responding to environmental variation. The extent and nature of the plasticity that evolves depends on the rate of environmental fluctuations and the capacity to track and respond to that variability. Reproductive environments represent one arena in which changes can be rapid. The finding that males of many species show morphological, physiological, and behavioral plasticity in response to premating and postmating reproductive competition (RC) suggests that plasticity is broadly beneficial. The developmental environment is expected to accurately predict the average population level of RC but to be a relatively poor indicator of immediate RC at any particular mating. Therefore, we predict that manipulation of average RC during development should cause a response in plasticity “set” during development (e.g., size of adult reproductive structures), but not in flexible plasticity determined by the immediate adult environment (e.g., behavioral plasticity in mating duration). We tested this prediction in *Drosophila melanogaster* males by manipulating 2 independent cues of average RC during development: 1) larval density and 2) the presence or absence of adult males within larval culture vials. Consistent with the prediction, both manipulations resulted in the development of males with significantly larger adult accessory glands (although testis size decreased when males were added to culture vials). There was no effect on adult plasticity (mating duration, extended mating in response to rivals). The results suggest that males have evolved independent responses to long- and short-term variation in RC.

## Lay Summary

We all respond to our social environment, to fit in or to become more competitive—fruitflies are no exception. Male larvae responded to their immediate and expected future competitive environments by developing larger sex accessory glands as adults. As expected, the larval competitive environment did not alter the extent to which adult males responded to their immediate sexual environment. The results show the multifaceted ways in which males respond to their sexual environments.

## INTRODUCTION

The level of reproductive competition (RC) experienced by males can be highly dynamic, as local sex ratios can vary over short temporal and small spatial scales (Kasumovic et al. 2008; Punzalan et al. 2010). When mating is costly and males can mate more than once, males are predicted to assess their short- and/or long-term competitive environment and adjust their reproductive effort accordingly (Parker et al. 1996, 1997). The magnitude of adjustments to reproductive effort predicted by these models depends on parameters such as the accuracy of information that males can perceive regarding the level of RC, resource availability, whether first or second matings are favored, and the extent of male control over mating frequency and duration (Parker and Pizzari 2010). In general, theory predicts increased investment with a higher risk of female remating, but diminishing investment as the number of rival males increases above 1 (Parker et al. 1996, 1997). Many studies demonstrate that males strategically allocate their resources by adjusting ejaculate size or content, and/or mating behaviors, according to RC cues (reviewed in Wedell et al. 2002; Bretman, Gage, et al. 2011). Empirical evidence suggests that males reduce investment in precopulatory behavior as RC increases (e.g., reduce courtship toward already-mated females) (Weir et al. 2011). However, once engaged in mating, males increase their investment in copulatory/postcoptulatory behaviors and in ejaculate investment in response to RC (Wedell et al. 2002; Bretman, Gage, et al. 2011).

Plasticity itself is diverse—it can be “fixed” or reversible, expressed in response to a critical environmentally sensitive period or to constantly fluctuating environments (Fusco and Minelli 2010). Likewise, the type of plasticity that males express in response to RC can vary according to the cues that are used and life-history stage at which cues are detected. For example, flexible and, to some extent reversible, behavioral plasticity in adults (e.g., mating duration) in response to immediate cues of adult RC such as presence of rival males, sex ratio skew, or female mating status is often observed (Bretman, Gage, et al. 2011). Irreversible plasticity in traits such as body and reproductive trait size can also be expressed and effectively “set” in response to conditions experienced or perceived during development. There are numerous examples of adult behavioral and developmental plasticity in response to RC cues across many taxa. Recent reviews cite 7 examples of developmental responses to sperm competition (SC) cues (Kasumovic and Brooks 2011) in comparison with >50 examples of adult responses via behavioral modulation (Bretman, Gage, et al. 2011). Whether responses to larval cues are less frequent or distinct, or simply understudied, is not yet clear. Examples of plasticity set during development include those in which individuals develop larger testes when raised in larger or more dense groups, for example, in the Indian mealmoth *Plodia interpunctella* (Gage 1995), the hermaphroditic leech *Helobdella papillornata* (Tan et al. 2004), the yellow dung fly *Scatophaga stercoraria* (Stockley and Seal 2001), and the cliff swallow *Petrochelidon pyrrhonota* (Brown CR and Brown MB 2003). In the dung beetle *Onthophagus taurus*, “major” males allocate more to condition but not to testis size when rivals are present (Simmons and Buzatto 2014). Hermaphroditic marine flatworms *Macrostomum lignano* also produce larger testes when raised in groups rather than pairs (Schaerer and Ladurner 2003). Males may also respond to cues from older cohorts. For example, male field crickets *Teleogryllus oceanicus* invest more in reproductive tissue (testis plus accessory gland [AG] mass) and condition (mass/size) following exposure as nymphs to the song of adult males (Bailey et al. 2010). Similarly, bank voles (*Myodes glareolus*) exposed to the odor of other males develop larger AGs (Lemaître et al. 2011). These studies demonstrate that larvae can use cues from their contemporaries as well as from previous cohorts to form the basis of their responses to anticipated future RC.

Responses in adult behavior to the immediate level of RC in the environment have been well described in *D. melanogaster*, where males exposed to rivals prior to mating subsequently mate for longer and achieve higher reproductive success in competitive and noncompetitive situations, in comparison with males held alone (Bretman et al. 2009). Exposure to rivals is associated with increased transfer of seminal fluid proteins (Wigby et al. 2009), a higher proportion of live sperm in the seminal vesicle (Moatt et al. 2014) and increased transfer of sperm (Garbaczewska et al. 2013). Extended mating duration in this context is under male control (Bretman et al. 2013a), is flexible, and can track changes in exposure to rival males over successive matings (Bretman et al. 2012).

There is growing interest in how different types of plasticity might interact (Fusco and Minelli 2010). Indeed, it has been suggested that socially cued behavioral plasticity (sensu Kasumovic and Brooks 2011) has 3 distinct forms that arise via different physiological, neural, and genomic mechanisms (Cardoso et al. 2015). The type and extent of plasticity that is selected will depend on how quickly the environment changes, how easily those changes can be tracked, and hence the magnitude of any potential fitness benefits. Hence, the capacity to phenotypically “match” developmental or adult plasticity to the appropriate prevailing environment is an important fitness component (Auld et al. 2010). When social and sexual environments are highly variable this matching may be easier to achieve for potentially reversible behavioral responses than it is for irreversible developmental plasticity set during development. For example, for adult behavioral plasticity expressed flexibly in response to short-term variation in the social environment, phenotype–environment matching is expected to be strong, even if the adult social environment varies rapidly and unpredictably. In contrast, individuals expressing developmental plasticity in response to cues sampled during development may suffer costs arising from poor phenotype–environment matching when adult environments vary (developmental instability costs sensu Dewitt et al. 1998). This idea also underlies recent thinking on the developmental origins of human disease (Gluckman and Hanson 2006). In the context of RC, we predict that developmental cues should accurately predict average levels of RC in the population overall, but be relatively poor indicators of immediate RC during adulthood.

Nevertheless, examples where the developmental environment affects behavioral plasticity on adulthood are known, for example, in *T. oceanicus* crickets that alter their satellite behavior (aggregation around another singing male) following exposure to male song as nymphs (Bailey et al. 2010). Likewise, preadult nutritional environment can affect adult reproductive strategies in burying beetles *Nicrophorus vespilloides*, whereby a period of starvation reduces male competitive ability but not parental care (Hopwood et al. 2013). If such anticipatory plasticity is achieved through epigenetic modifications, these could be maintained and expressed throughout life (Duncan et al. 2014), raising the possibility that the cues of RC during development and adulthood could be additive.

In this study, we used the *D. melanogaster* model system to test the prediction that manipulation of average RC by manipulations of the larval environment would alter developmentally determined, but not flexible adult behavioral plasticity. We manipulated cues of future RC during development and measured the effect on both developmental (testis, AG, and body size) and adult (mating duration and extended mating in response to rivals) plasticity. We manipulated the expected average level of RC during development by varying: 1) larval density at ad libitum food levels and 2) the presence or absence of adult males within the larval culture vials. Our aim with these manipulations of the developmental environment was to vary information about anticipated levels of average RC gained from within the cohort (density) and from previous cohorts (the presence of adult males in the culture vials). On the basis of the above prediction, we expected to see changes in developmentally plastic traits, but not in adult behavioral plasticity, in response to manipulation of the larval environment. We reasoned that if this prediction was incorrect and adult responses were also seen, it could indicate additivity in larval and adult cues of RC.

## MATERIALS AND METHODS

Fly rearing and all experiments were conducted in a 25 °C humidified room, with a 12:12h light:dark cycle. Flies were maintained in glass vials (75×25mm^2^) containing 8-mL sugar–yeast (SY) medium (Bass et al. 2007). Wild-type flies were from a large laboratory population originally collected in the 1970s in Dahomey (Benin), as used previously in our related studies (Bretman et al. 2009, 2010; Bretman, Westmancoat, et al. 2011; Bretman et al. 2012, 2013a, 2013b). Unless otherwise stated, larvae were raised at a standard density of 100 per vial. At eclosion, flies were collected and the sexes separated using ice anesthesia. Females and the males used to manipulate the larval environment (see below) were stored 10 per vial until use.

We conducted separate experiments to assess 2 different and independent cues of average future RC, to test whether both convey similar information and result in similar or distinct responses. We manipulated RC cues during development by varying 1) larval density and 2) the presence or absence of adult males in the larval culture vials. Manipulating these cues independently made it feasible to carry out large experiments to allow us to gain greater power for each test. To ensure ad libitum access to food despite increasing levels of resource competition and therefore to reduce gross effects on body size when manipulating larval density, we used SY medium (containing 150% of the standard yeast content; Bass et al. 2007) supplemented with yeast paste. We quantified developmentally determined plasticity responses to the manipulations of the larval environment by measuring body size, testis size, and AG size. We measured adult plasticity responses by maintaining adult males emerging from each of the 2 treatments above either alone or with a rival male prior to mating. The plastic response of mating duration to these manipulations was then recorded. We conducted 2 independent replicates (= blocks) of both experiments following exactly the same procedures.

### Responses of developmentally determined plasticity (body, testis, and AG size) to manipulations of the larval environment

To assess whether larvae could detect and respond to cues of average future RC within their own cohort, we raised larvae at a density of 20 or 200 larvae per vial, as described above. To assess whether larvae could detect and respond to cues of RC that are indicative of a previous cohort, larvae were raised at a standard density of 100 per vial (a standard density used in our related studies) but with or without 20 adult males present in the culture vials. Adult males were 5 days old when introduced to the larval culture vials, and they were removed the day before eclosion of the focal males.

We measured developmental plasticity in body, testis, and AG size for the adult males emerging from the larval density and adult male presence manipulations described above. At eclosion, 40 males from each treatment were frozen at −80 °C in batches of 10 for subsequent dissection and measurement of morphology. The individual data points were the mean of the measurements for the left and right side of each individual for each trait measured (body, testis, and AG size, respectively). If 2 measurements could not be made (e.g., through rupture of testes or AGs during dissection), the data were removed from further analysis. The length of the L3 wing vein was used as a robust proxy for overall body size (Gidaszewski et al. 2009) as it can be easily measured and shows high repeatability within individuals. We measured testis and AG perimeter after determining that these measures were reliable and repeatable (see below). For each male, wings were removed at their base using a scalpel and fixed between 2 glass slides. The males were then dissected in 20 μL of ice-cold phosphate-buffered saline. The testes and AGs were removed from the abdomen and placed flat on a microscope slide. To minimize rupturing of the reproductive structures, cover slips were not used. For all measurements, images were captured using an AxioCamMR5 camera on a Zeiss Stereo Discovery V12 microscope. A Plan Apo S 1.0× (60mm) objective was used to capture the wing measurements and an Achromat S 1.0× (63mm) objective to measure the testes and AGs. Images were analyzed using ImageJ software. To assess repeatability, a set of images of *N* = 30 males were captured, the testes and AGs then rearranged on the slide using a mounted needle, the images refocused, and a second set of images captured. These “before” and “after” measurements were tightly correlated (Pearson *r* > 0.9, *P* < 0.001 for testes and AGs). Hence, the measurements were highly repeatable. The final sample sizes for blocks 1 and 2 for the larval density manipulations were 30–39 and 32–33 and for the adult male manipulations 37–39 and 29–32, respectively.

### Responses of adult plasticity (mating duration) to manipulation of the larval environment

We tested whether manipulations of the larval environment, as described above, affected adult mating duration and the extent to which adult males extended mating duration following exposure to rivals (Bretman et al. 2009). We conducted a fully crossed design. For both larval manipulations (density or adult male presence), 80–120 males were either maintained singly for 4 days or exposed to rival males for 4 days. The rival males were reared under standard conditions as described above. All focal males were then given the opportunity to mate, and mating duration was recorded. Rival males were given a small wing tip clip for identification, using a scalpel under CO_2_ anesthesia. There is no evidence that wing clipping affects the ability of males to respond to rivals (Bretman et al. 2009). Virgin females were stored 10 per vial on medium supplemented with live yeast granules until the day before mating, when they were aspirated into individual mating vials. On the morning of the mating trial focal males were introduced to each of these vials and mating duration recorded. Pairs were given a 2-h period in which to mate and >90% of each groups mated within this time. The final sample sizes per treatment group were 48–59 for block 1 and 37–39 for block 2.

### Statistical analysis

Statistical analyses were performed in R v 2.14.0 (R Development Core Team 2013). For the 3 morphometric measurements, the mean of the left and right measures for each individual was used in all subsequent analysis. For all data sets analyzed, the data were not normally distributed and could not be normalized (Kolmogorov–Smirnoff tests *P* < 0.05). The effect of RC cues in the larval environment (larval density or presence/absence of adult males) on morphology was analyzed using generalized linear models (GLMs) with quasi-Poisson errors with larval cue treatment and block as fixed factors. Similarly, the effect on mating duration of larval environment and exposure to adult rivals was analyzed using GLMs with quasi-Poisson errors. Our reasoning for using Poisson errors was that mating duration was measured to the nearest minute (i.e., was discontinuous), and the data were non-normally distributed. The data also followed the other assumptions of a Poisson distribution, for example, they were bounded at a minimum of 5min, as mating durations of less than 5min were omitted from the data as they are considered “pseudomatings” in which there is no sperm transfer (Fowler 1973; Gilbert 1981). Mating duration also rarely extends beyond 20min, so the data have a larger variance in 1 direction. The use of the Poisson also allowed us to use quasi-errors for a more conservative test by accounting for over- or under-dispersion in the data. Factors were subtracted in turn from the maximal model using analysis of deviance. We present the results of these analyses, which allowed us to test the explanatory power of each variable in the model. We tested for relationships between morphological traits using Spearman rank correlations (Supplementary Figure S1). GLM analyses of testis perimeter and AG perimeter were conducted with and without including L3 wing vein length as a covariate in the models, to account for allometry.

## RESULTS

There was little evidence that any morphometric responses were affected by allometry, as there was only 1 significant pairwise correlation between the morphological traits measured (all *P* > 0.05, apart from block 2 of the density manipulation, where body size and AG size were positively correlated; Spearman’s rho = 0.246, *N* = 72, *P* = 0.037; Supplementary Figure S1). Hence, fitting quadratic functions did not explain more of the relationships in these analyses. In our tests for the effect of larval environment on male morphology described below, the results of analyses both with and without body size (L3 wing vein length) as a covariate were similar. This indicated that changes to reproductive morphology occurred independently of body size.

### Responses of developmentally determined plasticity (body, testis, and AG size) to manipulations of the larval environment

#### Effect of larval density manipulation

**Figure 1:**
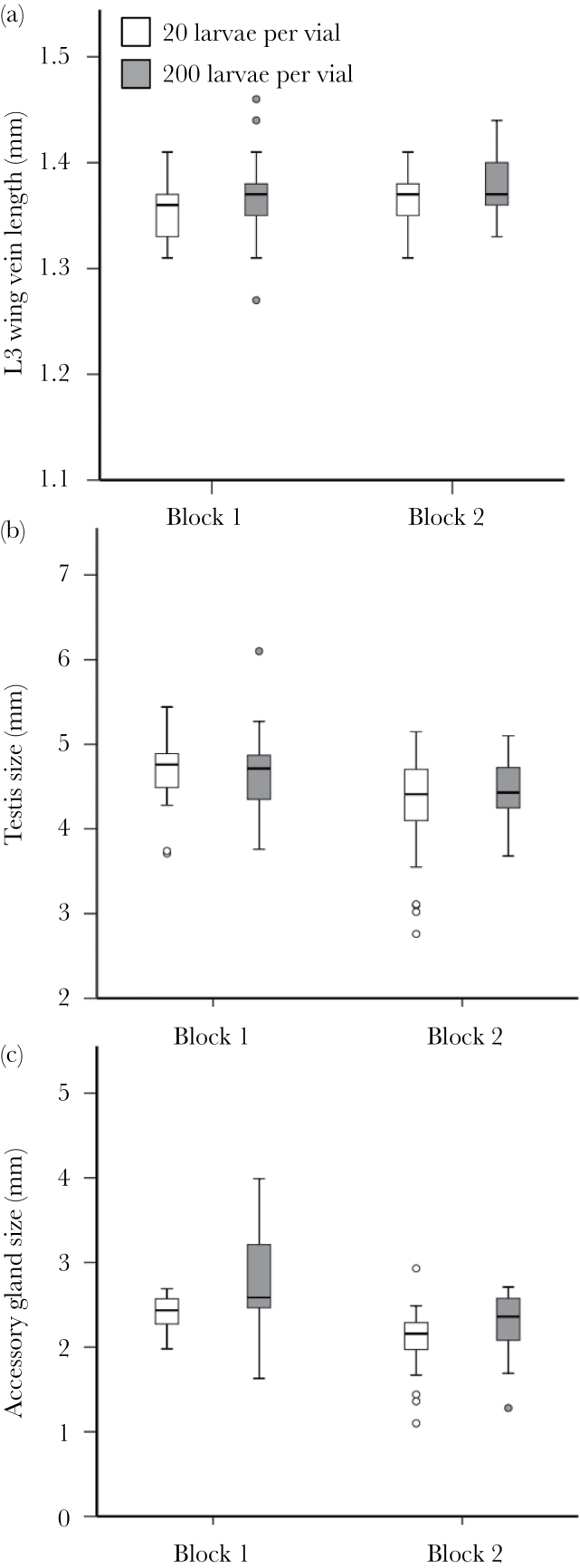
Boxplots showing the response of morphological traits to manipulation of larval density. Larvae were kept at low or high density (20 vs. 200 larvae per vial). Each experiment was replicated in 2 independent blocks. Morphological traits measured (in millimeters) were (a) body size (L3 wing vein length), (b) testis perimeter, and (c) AG perimeter. The data for testis and AG size are absolute values uncorrected for body size. White bars indicate low larval competition environments (20 larvae per vial); gray bars high larval competition environments (200 larvae per vial).

Males raised at higher density were significantly larger (analysis of deviance *F*_1, 144_ = 8.337, *P* = 0.004; Figure 1a), an effect that was consistent across experimental blocks (*F*_1, 144_ *=* 2.143, *P* = 0.145). There was no significant effect of larval density on testis size (without body size covariate *F*_1, 148_ = 0.648, *P* = 0.422, Figure 1b; with body size covariate *F*_1, 135_ = 0.774, *P* = 0.380). Males raised at higher density had significantly larger absolute (without body size covariate *F*_1, 150_ = 11.582, *P* **<**0.001; Figure 1c) and relative (with body size covariate *F*_1, 135_ = 9.752, *P* = 0.002) AG size. Here, there was significant variation between blocks, with males having generally smaller testis size (without body size covariate *F*_1, 148_ = 17.581, *P* < 0.0001, Figure 1b; with body size covariate *F*_1, 135_ = 16.816, *P* < 0.0001) and AG size (without body size covariate *F*_1, 150_ = 44.910, *P* < 0.0001, Figure 1c; with body size covariate *F*_1, 135_ = 42.880, *P* < 0.0001) in block 2. However, there was no interaction with the density treatment (all interactions *P* > 0.05). Hence, the different experimental blocks showed the same overall patterns in response to the manipulations of the larval environment.

#### Effect of presence of adult males in culture vials

**Figure 2:**
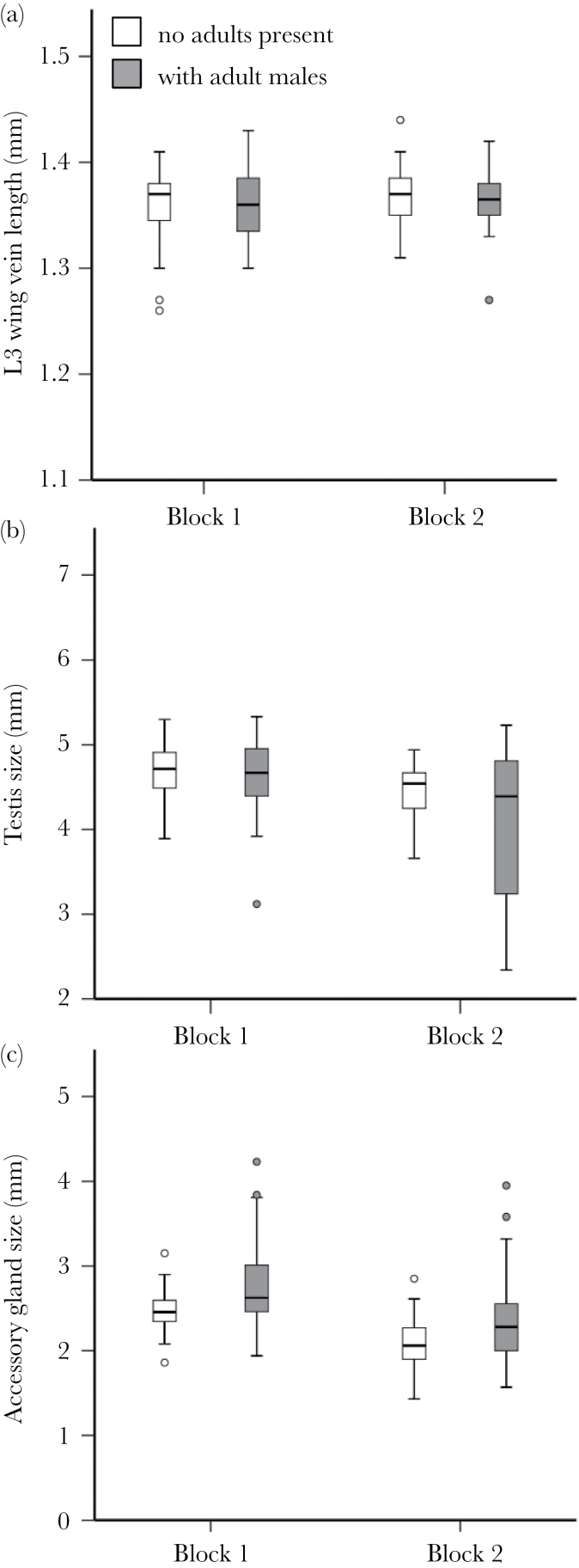
Boxplots showing the response of morphological traits to manipulation of adults in the larval environment. Larvae were kept at a standard density in the presence or absence of adult males in the culture vials. Each experiment was replicated in 2 independent blocks. Morphological traits measured (in millimeter) were (a) body size (L3 wing vein length), (b) testis perimeter, and (c) AG perimeter. The data for testis and AG size are absolute values uncorrected for body size. White bars indicate low competition environments (no adult males present in culture vials); gray bars high competition environments (adult males present in culture vials).

There was no effect of the presence or absence of adult males in the culture vials on the body size of males emerging from those cultures (*F*_1, 152_ = 0.144, *P* = 0.705; Figure 2a), with no significant difference between blocks (*F*_1, 152_ = 1.907, *P* = 0.169). Males developing as larvae in vials exposed to adult males had significantly smaller testis size (without body size covariate *F*_1, 144_ = 5.491, *P* = 0.020, Figure 2b; with body size covariate *F*_1, 138_ = 4.341, *P* = 0.038) and significantly larger AG size (without body size covariate *F*_1, 145_ = 20.101, *P* < 0.0001, Figure 2c; with body size covariate *F*_1, 145_ = 19.749, *P* < 0.0001). Again there was a significant main effect of block, with males having smaller testis size (without body size covariate *F*_1, 144_ = 19.990, *P* < 0.0001, Figure 2b; with body size covariate *F*_1, 138_ = 16.602, *P* < 0.0001) and AG size (without body size covariate *F*_1, 145_ = 36.991, *P* < 0.0001, Figure 2c; with body size covariate *F*_1, 145_ = 36.761, *P* < 0.0001) in block 2. However, the absence of any significant interactions between block and larval environment treatment (all interactions *P* > 0.05) showed that the responses to the larval manipulations were similar across the 2 blocks. There were no significant pairwise correlations between any of the traits measured in either block (all *P* > 0.20).

The results suggest that the manipulation of the larval environment by both methods led to significant responses in developmentally determined plasticity, particularly in AG size.

### Responses of adult plasticity (mating duration) to manipulation of the larval environment

**Figure 3:**
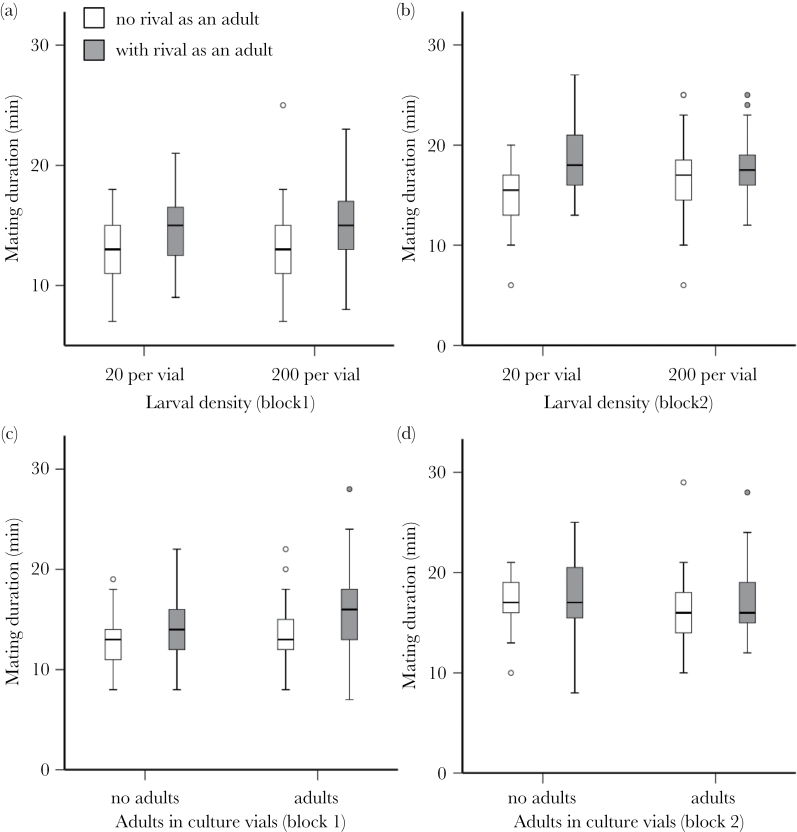
Boxplots showing the response of mating duration to manipulation of larval and adult environments. To manipulate cues of future RC, larvae were kept at low or high density (20 vs. 200 larvae per vial; a and b) or at a standard density in the presence or absence of adult males (c and d). To manipulate cues of RC in the adult environment, males were held during adulthood in isolation (white bars) or with another male (gray bars) for 4 days prior to the mating tests with virgin females during which mating duration (minutes) was recorded. Each experiment was replicated in 2 independent blocks (block 1, a and c; block 2, b and d).

There was no consistent effect of manipulating the larval environment on mating duration per se or the extent to which mating duration was extended in response to rivals. Larval density had no overall effect on mating duration (*F*_1, 368_ = 0.463, *P* = 0.497), although mating duration was generally longer in block 2 (*F*_1, 369_ = 86.685, *P* < 0.0001; Figure 3a and b). There was a significant interaction between experimental block and the effect of adult males in the culture vials on mating duration (*F*_1, 356_ = 5.496, *P* = 0.020; Figure 3c and d). In block 1, the “no adults” treatment mated for a shorter time overall than the “plus adults” treatment (mean ± standard error of 13.68±0.265min compared with 14.63±0.347min), whereas this was reversed in block 2 (no adults mean = 17.34±0.365min, plus adults = 16.68±0.386min). As expected based on our previous work (Bretman et al. 2009), males significantly extended their mating duration in response to rivals and hence perceived increases in the adult RC (in the larval density experiment *F*_1, 369_ = 34.068, *P* < 0.0001; adults in culture vials experiment *F*_1, 356_ = 17.284, *P* < 0.0001; Figure 3). There were no significant interactions between larval environment and adult environment for either larval manipulation (all *P* > 0.05), hence no evidence that larval environment affected the extent of adult plasticity.

The results suggest that the manipulation of the larval environment by 2 different methods had no effect on the expression of adult plasticity (mating duration and extended mating duration in response to the presence of rival males).

## DISCUSSION

The results were consistent with the prediction that manipulation of the larval environment should lead to responses in developmentally determined plastic traits but should have little or no effect on the expression of adult reproductive plasticity. Our main finding was that both types of manipulations of larval environment we employed had significant and repeatable effects on the morphology of fitness-related traits whose size is determined during development. At higher larval densities, males were significantly larger and had larger AGs (even after accounting for body size). Testis size was unaffected. When larvae were raised in the presence of adult males, body size was unaffected, but males developed significantly smaller testes and larger AGs (again independent from any allometric effects). Manipulations during development had no effect on adult mating duration. Mating duration was significantly modulated with respect to the presence or absence of a rival male during adulthood prior to mating, but the existence or magnitude of this effect was independent of the larval environment. Our findings support the idea that responses to larval environments that gave information on the average levels of RC were manifested through relatively fixed changes to the morphology of reproductive traits. There was no evidence that responses to larval RC interacted with the expression of adult behavioral responses to their immediate RC environment.

### Increased density and the presence of adult males caused a significant increase in AG size

AG size was sensitive to both of the manipulations of RC in the larval environment. This is line with previous findings that showed similar effects on reproductive tissue in total (Gage 1995; Bailey et al. 2010) or on AGs specifically (Lemaître et al. 2011). Of particular interest was our finding that larvae were sensitive to the presence of adult males in their culture vials. Indeed, they increased AG size to the same extent in response to information about RC of previous cohorts (presence of adult males) as they did to cues from their own cohort (larval density). The mechanisms by which larvae detect the presence of adults in the culture vials are as yet unknown. Nor is it yet apparent whether larvae are responding to the presence of adult males in the culture vials specifically, hence whether there might be different responses if adult females were used. Benefits to larvae of having adults added to the culture vials have previously been reported. Wertheim et al. (2002) found that the presence of adults led to increased larval survival, and in some cases increased adult body size. They attributed this effect to the transfer of beneficial yeasts to the food by the adults that were present, a suggestion supported by subsequent studies (Stamps et al. 2012). These studies raised flies on natural fruit diets poor in yeast, in which the effect of any nutrient supplementation might be particularly evident. In this study, we used an artificial diet with extra live yeast added and did not find any effect of the presence of adults on body size. Hence, nutritional transfer from adults seems an unlikely explanation for the observed changes to reproductive morphology. Previous studies have shown AG size to be a key fitness-related trait. Male *D. melanogaster* with larger AGs gain significantly more offspring when in competition, and large AG size is associated with significantly increased pre- and post-reproductive success (Bangham et al. 2002; Wigby et al. 2009). AG size is also a key limiting factor in a male’s overall reproductive success, as across successive matings, loss of fertility is associated with size of AGs rather than testes (Linklater et al. 2007). Direct selection for larger AGs is associated with significantly increased production and transfer of at least one ejaculate component (sex peptide), as well as significantly increased paternity under strong RC (Wigby et al. 2009). Collectively, this body of data indicates that AG size is a fundamentally important fitness-related trait. The strong and replicated response in AG size following both types of manipulations of the larval environment in this study, together with the previous research demonstrating a positive correlation between AG and fitness, suggests that plasticity in AG size, mediated by indices of RC, is adaptive.

Not all manipulations of RC via larval density in *D. melanogaster* appear to result in fitness benefits or variation in AG size or activity consistent with the patterns we observed here. For example, increased larval density has also been reported to lower a male’s success in SC in matings with already-mated females (Amitin and Pitnick 2007). McGraw et al. (2007) also manipulated larval density and found no effect on the transcription of 9 AG seminal fluid proteins tested. More recently, it was reported that males reared at higher larval densities produced lower amounts of 2 seminal fluid proteins (sex peptide and ovulin) but transferred relatively more sex peptide during mating Wigby et al. (2015). This was interpreted as condition-dependent strategic ejaculation in response to mating opportunities, as high larval density-reared males were smaller and less successful at gaining matings. However, as acknowledged, this cannot currently be disentangled from the possibility that higher adult RC is signaled by increased larval encounter rate. As discussed below, in our study, we found instead that increasing larval density increased body size. This suggests that body size was not determined by nutritional limitation and that by using a rich diet we manipulated encounter rate specifically. It would therefore be informative to test whether males developing larger AGs under crowded, but not resource-limited, conditions produce or transfer more seminal fluid proteins. Together, these findings suggest that direct tests of the fitness effects of plasticity in AG size in response to larval SC cues using multiple fitness measures would be useful.

The finding that larvae responded to increased RC, as signaled by the presence of adults in the culture vials and hence indicative of RC in the previous generation, is perhaps surprising finding given that in species with short generation times we expect the strongest competition to derive from within rather than between cohorts. However, our finding is consistent with some previous reports. For example, bank voles exposed to the odor of sexually mature males develop larger AGs (Lemaître et al. 2011). Likewise, male *T. oceanicus* develop larger reproductive organs (using a combined measure of testis and AG size) following exposure to the song of adult males as nymphs (Bailey et al. 2010). Responses to both current and previous cohorts could be beneficial if there is generational overlap and if individuals mature asynchronously (Kasumovic and Brooks 2011). Flies in our large cage populations from which the experimental individuals were derived may fulfill these criteria—they reproduce asynchronously throughout the year. This may mirror the situation in many natural populations (David et al. 1984). Hence, generational overlap and direct competition between previous and current cohorts may occur. An informative comparison would be with populations that have discrete generations, where responses to indicators of between-cohort competition are not expected to be selected for. Future work could investigate these possibilities.

### Testis size does not respond to larval density but is reduced in the presence of adult males

Different reproductive organs can respond independently to cues of RC. For example in bank voles, AGs, but not testes, were sensitive to cues of future RC (Lemaître et al. 2011). Nevertheless, our finding that testis size decreased in the presence of adults was unexpected. We found no evidence that this represented a trade-off in tissue development, as we found no negative correlation between testis and AG size. Such trade-offs have been documented, as in the negative relationship between testis and horn size in *Onthophagus binodis* dung beetles (Simmons et al. 1999) and testis and mandible size in *Hemideina crassidens* wetas (Kelly 2008). It is possible that there were trade-offs with other tissues that we did not measure.

Our finding that testis size did not covary with larval density is in contrast to previous studies in other species (Gage 1995; Stockley and Seal 2001; Brown CR and Brown MB 2003; Tan et al. 2004) and with the idea that sperm quality and number is responsive to adult cues of competition in *D. melanogaster* (Garbaczewska et al. 2013; Moatt et al. 2014). Further data suggesting that testis size is an important correlate of SC comes from studies in which testis size was observed to decrease in experimental evolutionary studies that enforced monogamy (e.g., in *S. stercoraria* [Hosken and Ward 2001] and *O. taurus* [Simmons and García-González 2008]). Indeed, *M. ligano* raised in groups show increased testis size and sperm production, suggesting that sperm production efficiency also increases under increased SC (Schaerer and Vizoso 2007). In addition, there is also some debate as to whether testis size is responsive to numerical SC or to the number of mating opportunities (Vahed and Parker 2012). Variation in larval density potentially manipulates both of these selective pressures to them to different degrees, potentially explaining some of the discrepancies between studies. In *D. melanogaster*, males evolved larger testes in experimental evolution lines maintained under female biased conditions, in which SC is low and mating frequency high (Reuter et al. 2008). Likewise in *Drosophila pseudoobscura*, selection lines held under a regime of promiscuity evolved larger AGs but not testes, in comparison with monandrous lines (Crudgington et al. 2009). Hence, testis size, and potentially sperm number, may be relatively insensitive to SC levels per se, consistent with the finding that testis size shows no association with either pre- or post-copulatory competitive success in *D. melanogaster* (Bangham et al. 2002). Taken together these data suggest that in *D. melanogaster*, AG size and seminal fluid production show closer associations with SC than do testis size/sperm number. Alternatively, AG size may express more plasticity and/or have fewer constraints than testis size.

### Manipulations of larval density altered adult body size

In our experiments, we manipulated larval density under ad libitum conditions within a range of densities to which morphology is relatively insensitive (Edward and Chapman 2012). We did this in an effort to increase or decrease encounter rates between larvae while equalizing food availability. Although we cannot directly rule out the possibility that larvae responded to competition in general, rather than specifically to social cues of future RC, changes in AG size were independent from body size and thus did not result solely because of a scaling relationship. The evidence that food supply was not limiting was the absence of a decrease in body size as larval density increased, in contrast to the pattern found in numerous previous studies (Lazebnyi et al. 1996; Byrne and Rice 2006; Amitin and Pitnick 2007; Rode and Morrow 2009; McNamara et al. 2010; Durisko and Dukas 2013; Wigby et al. 2015). The discrepancies can be reconciled by the realization that larval density can be manipulated under food limiting or ad libitum conditions, leading to divergent outcomes. Indeed, the increase in body size suggests that, given unlimited food, a response to increased density is to develop a larger body that will be more successful in direct competition with rivals for matings with females. This supports the idea that social competition for resources can maintain heritable genetic variation in fitness-related traits (Wolf et al. 2008).

It is becoming increasingly evident that, despite the potential for increased resource competition, fruit fly larvae often aggregate (Durisko and Dukas 2013). Larvae in aggregations initiate burrowing activity faster, which has been suggested as a strategy to avoid parasitism (Rohlfs and Hoffmeister 2004) or a way of more rapidly finding a food-free pupation site (Wu et al. 2003). Larvae are reported to aggregate more on hard food substrates, perhaps suggesting that higher densities could render food more accessible (Durisko et al. 2014). This is consistent with our finding of increased size at higher densities.

A related issue is whether development time is affected. Studies in *D. melanogaster* (Wertheim et al. 2002) and in almond moths (*Cadra cautella*) (McNamara et al. 2010) found those raised at higher densities took longer to develop and were smaller than those raised at lower densities. We did not notice any gross differences in the timing of adult collections, but there could of course be finer scale differences. In general, the relationship between size and development time can vary, with some studies finding larger flies take longer to develop (e.g., Partridge and Fowler 1993) and others detecting no relationship (e.g., Santos et al. 1997).

Whether the body size differences in response to density that we detected have any knock-on fitness consequences remains to be determined. In *D. melanogaster*, larger males are reported to deliver more courtship (Partridge et al. 1987) and body size also predicts both pre- and post-copulatory success (Bangham et al. 2002; Wigby et al. 2015). Hence, elevated RC cues perceived during development are expected to increase body size. Our results were consistent with this prediction. However, the overall relationship between male body size and fitness is complex, as larger males produce more courtship and receive more matings, but reduce female lifespan and offspring survival (Friberg and Arnqvist 2003).

### Adult behavioral plasticity did not respond to manipulations of RC during development

Effects of juvenile social environment on adult behavior have been reported as discussed earlier (e.g., Bailey et al. 2010). However, here we found no effect of larval environment on adult behavior measured as mating duration. Instead, mating duration was modulated solely according to immediate changes in the adult environment (presence or absence of rival males). There was also no evidence that the manipulations of the developmental environment affected the magnitude of the response of adults to rival males.

Together, the data suggest that *D. melanogaster* males have multiple strategies to respond adaptively to variation in RC. During development, they respond plastically to cues that give information about the likely average level of RC expected within their population. They can then match their reproductive morphology accordingly, developing larger AGs if there is high RC. These plastic responses are, however, characterized by early commitment to a fixed and irreversible strategy (e.g., large AGs for a high average RC and vice versa). In contrast, adult males make fast and flexible behavioral (and physiological) adjustments to track short-term variation in RC. The amount of RC during a particular mating cannot accurately be predicted by the developmental environment, providing an explanation for why adult behavioral traits are relatively unresponsive to those cues.

The existence of plastic strategies implies that there are underlying trade-offs, otherwise such allocation decisions would not be necessary. We have previously found that males maintained with rivals throughout their life suffer decreased lifespan and late-life mating success, implying that adult responses to rivals carry significant costs (Bretman et al. 2013b). Previous work in Lepidoptera (Gage 1995) showed that larvae raised at higher densities produced larger testes but had decreased lifespan, perhaps reflecting developmental costs. We did not find any direct trade-offs between the morphological traits that we measured, hence there was no evidence for a negative correlation between AG and testis size, as suggested in previous studies (Nijhout and Emlen 1998). However, developmental plasticity is predicted to exact higher costs than behavioral plasticity, both in terms of production and in phenotype–environment mismatches (Dewitt et al. 1998; Auld et al. 2010). Given this prediction, it is noteworthy that larvae responded to future RC using information from both their own and older cohorts, suggesting that both types of cues contain reliable information. It would, however, be informative to test whether there are differences in fitness costs of mismatches between predicted and actual RC environments arising from incorrect developmental versus adult cues. It would also be interesting to compare the costs of AG size modulation with those of plasticity in behavior or ejaculate composition. Overall, our results show that RC is a powerful evolutionary driver, with the potential to select strongly and potentially independently on both developmental plasticity and behavioral strategies. Males therefore use multiple facets to respond to the average level of SC in the population, as well as to immediate short-term, SC environments.

## SUPPLEMENTARY MATERIAL

Supplementary material can be found at http://www.beheco.oxfordjournals.org/

## FUNDING

The study was supported by research grants from the BBSRC (BB/H002499/1, BB/H008047/1) and NERC (NE/J024244/1) to T.C., M.J.G. Gage, and A.B. C.F. is supported by an Emmy Noether Fellowship of the DFG and AB by a University of Leeds Academic Fellowship.

## Supplementary Material

Supplementary Data
