# Supplementary material for: Effect of competitive cues on reproductive morphology and behavioral plasticity in male fruitflies
Source: Behav Ecol. 2015 Oct 25;27(2):452–61. doi: 10.1093/beheco/arv170 (PMC4797378; doi:10.1093/beheco/arv170)
Supplement: Supplementary Data [file supp_27_2_452__index.html]

Effect of competitive cues on reproductive morphology and behavioral plasticity in male fruitflies — Effect of competitive cues on reproductive morphology and behavioral plasticity in male fruitflies — Supplementary Data 

# Effect of competitive cues on reproductive morphology and behavioral plasticity in male fruitflies

## Supplementary Data

Data files

- Supplementary Data - Supplementary Data
