# Supplementary figures and images for: Effect of competitive cues on reproductive morphology and behavioral plasticity in male fruitflies
Source: Behav Ecol. 2015 Oct 25;27(2):452–61. doi: 10.1093/beheco/arv170 (PMC4797378; doi:10.1093/beheco/arv170)

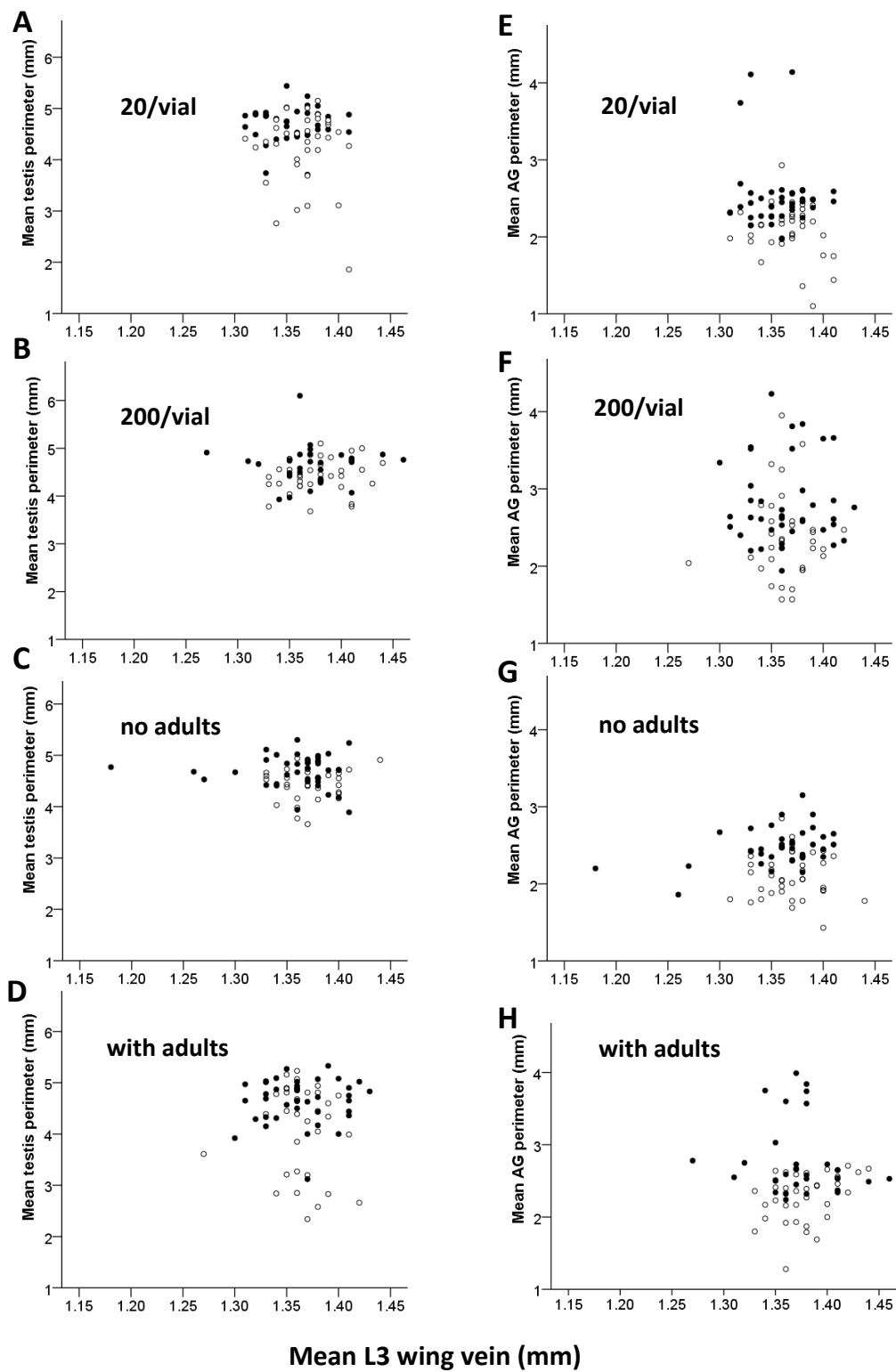

**Figure S1**

Supplement: Supplementary Data [file supp_arv170_LAA_ms_supp_mat_figs_FINAL.pdf]
